# Deep learning for HGT insertion sites recognition

**DOI:** 10.1186/s12864-020-07296-1

**Published:** 2020-12-29

**Authors:** Chen Li, Jiaxing Chen, Shuai Cheng Li

**Affiliations:** Department of Computer Science, City University of Hong Kong, Kowloon, Hong Kong SAR, HongKong, China

**Keywords:** Deep residual model, HGT insertion site, DNA sequence feature

## Abstract

**Background:**

Horizontal Gene Transfer (HGT) refers to the sharing of genetic materials between distant species that are not in a parent-offspring relationship. The HGT insertion sites are important to understand the HGT mechanisms. Recent studies in main agents of HGT, such as transposon and plasmid, demonstrate that insertion sites usually hold specific sequence features. This motivates us to find a method to infer HGT insertion sites according to sequence features.

**Results:**

In this paper, we propose a deep residual network, DeepHGT, to recognize HGT insertion sites. To train DeepHGT, we extracted about 1.55 million sequence segments as training instances from 262 metagenomic samples, where the ratio between positive instances and negative instances is about 1:1. These segments are randomly partitioned into three subsets: 80% of them as the training set, 10% as the validation set, and the remaining 10% as the test set. The training loss of DeepHGT is 0.4163 and the validation loss is 0.423. On the test set, DeepHGT has achieved the area under curve (AUC) value of 0.8782. Furthermore, in order to further evaluate the generalization of DeepHGT, we constructed an independent test set containing 689,312 sequence segments from another 147 gut metagenomic samples. DeepHGT has achieved the AUC value of 0.8428, which approaches the previous test AUC value. As a comparison, the gradient boosting classifier model implemented in PyFeat achieve an AUC value of 0.694 and 0.686 on the above two test sets, respectively. Furthermore, DeepHGT could learn discriminant sequence features; for example, DeepHGT has learned a sequence pattern of palindromic subsequences as a significantly (*P*-value=0.0182) local feature. Hence, DeepHGT is a reliable model to recognize the HGT insertion site.

**Conclusion:**

DeepHGT is the first deep learning model that can accurately recognize HGT insertion sites on genomes according to the sequence pattern.

## Background

Horizontal Gene Transfer(HGT) [1] refers to the sharing of genetic materials between distant species that are not in a parent-offspring relationship [2]. HGT allows different species to share genomic fragments, thus creating a complex network among different species [3]. It is the fundamental mechanism for the spread of antibiotic resistance in bacteria [4, 5] and plays an important role in the evolution of bacteria [6–8]. Conjugation [9], transformation [10], and transduction [11] are the three most recognized mechanisms for HGT. Conjugation requires physical contact between a donor cell and a recipient cell. Then the genetic material, such as conjugative transposons [12], is transferred through plasmids. Transformation is the uptake of foreign genetic material from the surrounding environment and is relatively common in bacteria. Transduction is mainly mediated by phage and could occur more than 1,000 times in specific environments [13]. Through these mechanisms, functional unit of DNA, such as operon [14], and mobile genetic elements [15], such as transposons containing antibiotic resistance genes, could be incorporated into the genome of recipients [12]. Therefore, HGTs improve the bacteria’s ability to adapt to changing environments. HGTs are often observed and well studied in prokaryotes. Recent research demonstrates that around 80% of genes in prokaryotes were involved in HGT at some point in their history [16]. HGTs could also occur between prokaryotes and eukaryotes [17]. From prokaryotes, eukaryotes acquire genes that are helpful to detoxify novel environments [18–20]. Moreover, through HGT, many eukaryotes benefit from the acquisition of genes encoding biosynthetic enzymes to live in extremely nutrient-poor environments [21, 22].

Mobile genetic elements (MGE), such as transposons, are the main agents of HGT [15]. Existing research on transposons demonstrates that the transposon ends usually have special sequence features, such as inverted repeats [23], AT-rich [12], etc. These sequence features make transposon easily transferred across cells by plasmids, phage, or integrative conjugative elements (ICEs). Other agents of HGT may also have specific sequence features [24]. These facts make it possible to recognize HGT insertion sites according to the sequence features at the sites. Deep learning is such a powerful method to extract features from DNA sequences. It is a class of machine learning algorithms based on artificial neural networks. It allows computational models composed of multiple processing layers to learn representations for data with multiple levels of abstraction [25]. Starting from 2012, deep learning has achieved great performance breakthroughs in computer vision [26, 27], speech recognition [28], and so on. More recently, deep learning was adopted to process DNA sequence data and Convolutional Neural Networks (CNN) is the most wildly used deep learning model in the field of bioinformatics. In 2015, [29] proposed DeepBind to predict DNA and RNA-binding proteins based on in vitro and in vivo assays. It performs better than the state-of-the-art methods from the DREAM5 in vitro TF-DNA motif recognition challenge [30]. Zhou and Troyanskaya [31] developed DeepSEA to predict chromatin effects of sequence alterations with single-nucleotide sensitivity by learning regulatory sequence codes from large-scale chromatin-profiling data. Researchers also have used CNN models to predict functional elements in the genome, such as promoter [32] and enhancer [33]. These applications imply that deep learning could effectively learn features from raw DNA sequences to perform the classification task. This motivates us to propose a deep learning model, named DeepHGT (https://github.com/lichen2018/DeepHGT), which learns sequence features to recognize HGT insertion sites on reference sequences.

In order to train DeepHGT, we should get DNA sequences at HGT insertion sites. We utilize a traditional alignment tool LEMON [34] which is based on split reads re-alignment [35] and DBSCAN [36] to detect and label HGT insertion sites. Then we could collect DNA sequences at the detected HGT sites. In order to prove the specialty of DeepHGT, we also compare its performance with other machine learning models implemented in PyFeat [37]. PyFeat generates features from DNA sequences to train machine learning models. The generated features include zCurve, gcContent, ATGC ratio, Cumulative Skew, Chou’s Pseudo composition, gap-based K-mer frequency, and so on. These features could capture the frequency distributions of various permutations of the base nucleotide in the sequences [37].

As described in the “Methods” section, by utilizing LEMON we collect a set of 1,556,694 sequence segments from 262 metagenomic samples [38]. 50% of the set are positive samples that are extracted at HGT insertion sites on reference genomes. The remaining sequences are negative samples. The set is randomly partitioned into three subsets: 80% of them as the training set, 10% as the validation set, and the remaining 10% as the test set. DeepHGT has achieved Area under the Curve of ROC (AUC) value of 0.8782 and Average-Precision (AP) value of 0.899 in the test set. Compared to the performance of four machine learning models implemented in PyFeat, features learned by DeepHGT are more discriminant than those generated by PyFeat and make DeepHGT achieve better performance. Besides, 125 correctly classified positive test sequences at HGT insertion sites contain palindromic subsequences. For each sequence, any continuous subsequence can be treated as a local feature. We define HGT-Index to measure the contribution of its local feature to the prediction value of the sequence. Statistic test results demonstrate that palindromic subsequences, which are typical sequence patterns in MGE, are significantly local features. In addition, to further evaluate the generalization of DeepHGT, we obtain an independent test set of 689,312 sequence segments from 147 metagenomic samples [39] using LEMON. The ratio between positive and negative samples is 1:1. DeepHGT has achieved the AUC value of 0.8428 and the AP value of 0.8743, which supports the good generalization of DeepHGT. So DeepHGT can accurately recognize HGT insertion sites on genomes according to sequence pattern.

## Results

### Percentage distribution of positive samples at species/genus level

The 778,347 positive sequences extracted from 262 metagenomic samples belong to 3,070 species and 711 genera. Table 1 summarized the percentage distribution of the top 10 most abundant species/genera. As we can see, *Microbacterium esteraromaticum* is the species to which the greatest number of positive sequences belong. Its percentage is only 13.13%. The percentages of the other nine species are less than 10%. Furthermore, we calculate the percentage distribution of the 3,070 species. Its standard variance is 0.30%. *Microbacterium* is the genus to which the greatest number of sequences belong. Its percentage is 14.38%. The standard variance for the percentage distribution of the 711 genera is 0.92%. Therefore, sequences are evenly distributed across the 3,070 species and 711 genera. In another word, sequences are not enriched to a small number of species/genus. Therefore, the dataset is balanced at the appropriate species/genus level. Since this dataset is mainly used to train and validate DeepHGT, we call this dataset as the positive training dataset. The 344,656 positive sequences extracted from 147 metagenomic samples in the independent test set belong to 2,139 species. Appendix Table 7 compares the two percentage distributions of the top 10 most abundant species to which sequences in the positive training dataset and the independent positive test dataset belong. As we can see sequences in the two datasets have very different composition at species level.

**Table 1:** Percentage distribution of Top 10 most abundant species/genera to which positive samples belong

Top 10 Species	Percentage (%)	Top 10 Genera	Percentage (%)
Microbacterium esteraromaticum	13.13	Microbacterium	14.38
Mycolicibacterium monacense	7.36	Bacteroides	12.35
Mycobacterium sp. 852002-51961_SCH5331710	3.08	Bifidobacterium	8.00
Faecalibacterium prausnitzii A2-165	2.39	Mycolicibacterium	7.73
Collinsella aerofaciens ATCC 25986	1.97	Mycobacterium	6.21
Collinsella sp. 4_8_47FAA	1.94	Collinsella	5.89
Gemmiger formicilis	1.69	Clostridium	3.23
Collinsella sp. TF06-26	1.64	Faecalibacterium	2.70
Bifidobacterium longum	1.55	Alistipes	2.49
Bacteroides caccae	1.50	Roseburia	2.37

### Overview of DeepHGT

**Fig. 1:**
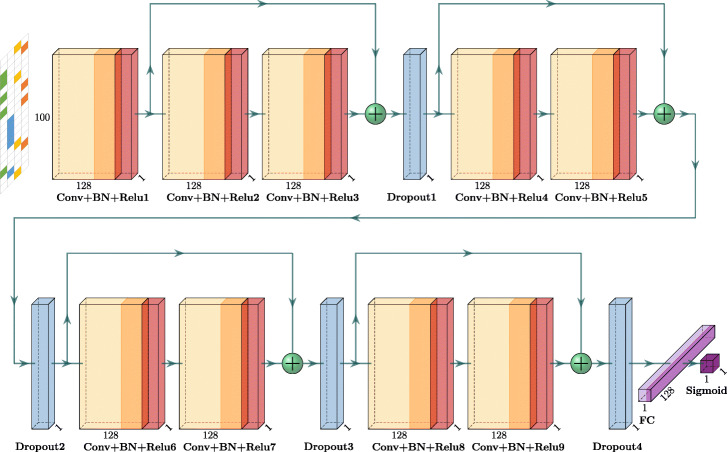
Overview of DeepHGT

We propose DeepHGT as illustrated in Fig. 1. DeepHGT is a deep residual neural network [40], which contains four residual blocks. Each residual block contains two *Conv+BN+Relu* sub-blocks and one skip-connection, which directly connects the input and output of the residual block, here *Conv* denotes the Convolutional layer, *BN* denotes the Batch Normalization layer [41], and *Relu* denotes the ReLU activation layer. In general, as we increase the number of layers in the neural network, its performance on both training and test data will decrease, this is called the degradation problem [40]. By adding skip-connection to skip some layers, the residual neural network is equal to the integration of multiple neural networks with different depths. This solves the degradation problem and makes the residual neural network go deeper to extract more mid-level and high-level features than shallow models. These extra features also enable the residual neural network to achieve better performance than shallow models. In order to improve the generalization performance of DeepHGT, we add one Dropout layer [42] behind each residual block. Dropout is an efficient trick to reduce overfitting during training. By randomly dropping hidden nodes, the training process is equivalent to training a large number of neural networks with different architectures in parallel. This makes DeepHGT learn more robust features thus better generalize to new data.

DeepHGT is implemented by using Keras and contains 2,119,297 trainable parameters. We set the length of the input sequence as 100 [43] and convert each sequence to a 100×4 matrix using the one-hot encoding method, where each position corresponds to a four-element vector with one nucleotide’s bit set to one [33]. All convolutional layers in DeepHGT have the same number of filters 128 and the same kernel size 4×1 with slide step 1. The first two Dropout layers have the same dropout rate 0.1, the dropout rate of the third Dropout layer is 0.25, and the dropout rate of the last Dropout layer is 0.5. By setting the dropout rate small in lower Dropout layers, we could maintain most low-level features. The large dropout rate in higher Dropout layers is helpful for learning useful high-level features. Right behind the last Dropout layer is a fully connected layer, which contains 128 units. Since our task is a binary classification problem, the output layer is a *Sigmoid* function. We don’t apply the pooling layer in DeepHGT since we found that pooling layers reduce the spatial dimensions of feature vector by a factor of 2, which leads to the loss of too much feature information and decreases the performance of DeepHGT in experiments.

### DeepHGT predicts HGT insertion sites

We set the batch size as 120 and utilize Stochastic Gradient Descent (SGD) to minimize the objective loss function of DeepHGT. The learning rate is 0.001. Since our task is to predict whether the input sample is positive, we set the loss function as binary cross-entropy. The number of epochs for training is 2,900. By changing the number of residual blocks in DeepHGT, we get other four deep learning models as comparisons. Their performance is measured by AUC and precision-recall curve. The precision-recall curve shows the tradeoff between precision and recall for different thresholds and Average-Precision (AP) is the weighted mean of precisions achieved at each threshold.

**Fig. 2:**
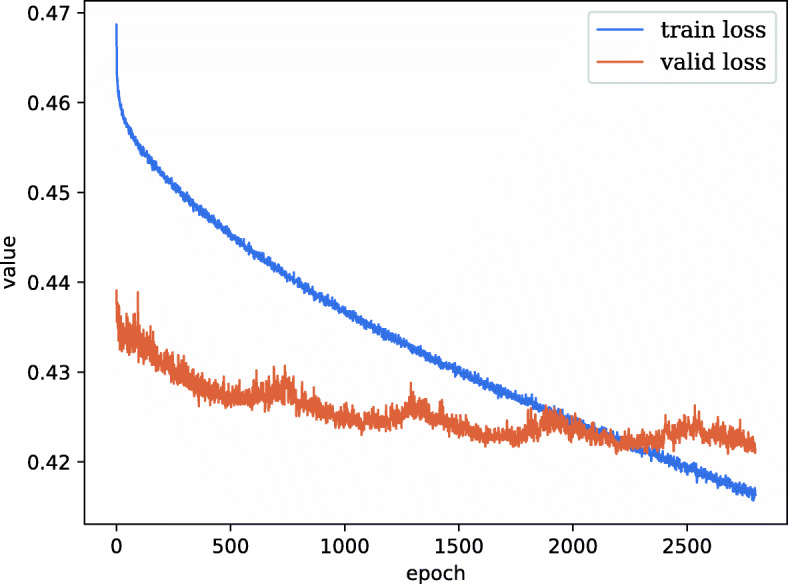
Training and validation loss evolution during the training of DeepHGT

The set of 1,556,694 sequence is randomly partitioned into three subsets: 80% of them as the training set, 10% as the validation set, and the remaining 10% as the test set. The DeepHGT is trained on one NVIDIA Tesla V100 GPU. Figure 2 illustrates the evolution of training and validation loss during the training process of DeepHGT. During the first 200 epochs, both of train loss and validation loss decrease fast, this demonstrates that DeepHGT efficiently learns useful sequence features from the training dataset to distinguish positive and negative samples. Since we have utilized data augmentation methods on the training set and not on the validation set, this makes the training set become more diverse and contain more hard samples to train. Therefore, the training loss is larger than the validation loss during 2,000 epochs. After 2,200 epochs, the validation loss fluctuates around 0.423, while the training loss continues to decrease rapidly and become lower than validation loss. This is because DeepHGT gets stuck in a local minimum, and continuing training makes DeepHGT overfit the training data without achieving better performance on the validation set. Therefore, we stop the training process after 2,900 epochs.

**Fig. 3:**
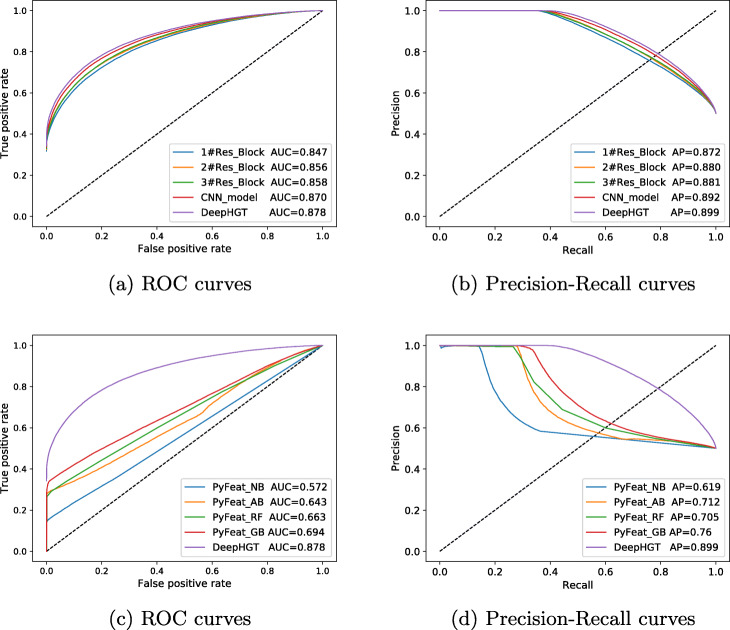
**a** and **b** compare ROC and Precision-Recall curves of DeepHGT and the other five deep learning models. DeepHGT has achieved the highest AUC value 0.878 and AP value 0.899. **c** and **d** compare the performance of DeepHGT and four machine learning models implemented in PyFeat: Naive Bayes (PyFeat_NB), Adaboost Classifier (PyFeat_AB), Random Forest (PyFeat_RF), and Gradient Boosting Classifier (PyFeat_GB)

Figure 3a and b illustrate the Receiver OperatingCharacteristic (ROC) and Precision-Recall curves of DeepHGT and the other five deep learning models with different architectures. 1#Res_Block denotes that the deep residual model contains 1 residual block, 2#Res_Block denotes that the deep residual model contains 2 residual blocks, and so on. CNN_model has the same number of convolutional blocks as DeepHGT without skip-connections. DeepHGT contains 4 residual blocks as shown in Fig. 1. DeepHGT has achieved the highest AUC value of 0.8782 and the AP value of 0.8994. As we can see, by increasing the number of residual blocks, the deep learning model achieves better performance, which means that deeper models can learn more high-level features and get better generalization than shallow models. DeepHGT has better performance than CNN_model, which validates skip-connections are useful to prevent overfitting. Therefore, designing deep learning models with proper architectures is important to achieve good performance. Figure 3c and d compare the performance of DeepHGT and four machine learning models implemented in PyFeat including Naive Bayes [44] (PyFeat_NB), Adaboost Classifier [45] (PyFeat_AB), Random Forest [46] (PyFeat_RF), and Gradient Boosting Classifier [47] (PyFeat_GB). PyFeat extract features from training and test datasets. The features are then used to train and test the four machine learning models. Their parameters are set in PyFeat. Compared to the four models in PyFeat, DeepHGT has achieved the best performance, although PyFeat_GB has achieved AUC value 0.694 and AP value 0.76 based on the features extracted by PyFeat. Table 2 compares the accuracy of DeepHGT and other methods. DeepHGT achieved the highest accuracy score of 0.794. Therefore, sequence features learned by DeepHGT are more general and efficient than those extracted by PyFeat. And this also makes DeepHGT achieve better classifier performance than the other four models in PyFeat.

**Table 2:** Comparison of accuracy of DeepHGT and other methods

1#Res_Block	2#Res_Block	3#Res_Block	CNN_model	DeepHGT
0.761	0.772	0.773	0.781	0.794
PyFeat_rf	PyFeat_ab	PyFeat_gb	PyFeat_nb	DeepHGT
0.597	0.636	0.653	0.500	0.794

We then perform the Pairwise Delong test on AUCs of these models. The null hypothesis is that two ROC curves have the same AUC values. Small *p*-value denotes two AUC values are significantly different, which means the two models have significantly different performance. As illustrated in Appendix Table 8, all pairwise Delong test results have a *p*-value of less than 0.05, which means all models have significantly different performance.

### DeepHGT predicts HGT insertion sites in different species

**Fig. 4:**
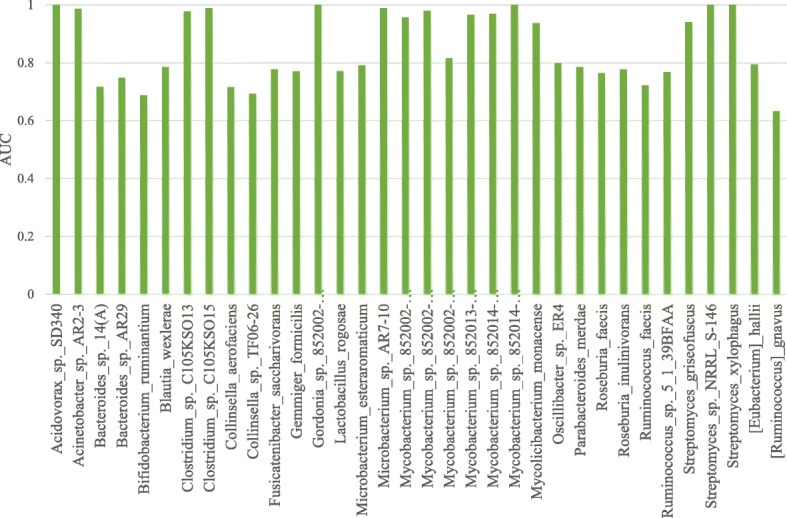
AUC values of DeepHGT on predicting HGT insertion sites in different species

The test dataset consists of samples from several species, such as *Streptomyces griseofuscus*, *Oscillibacter sp. ER4*, *Roseburia inulinivorans*, *Acidovorax sp. SD340*, etc. We divide positive test samples into subsets based on species to which samples belong. Since each subset contains only positive samples from the same species, we randomly extract the same number of negative samples from reference sequences of the species. We then use these test subsets to evaluate the AUC values of DeepHGT on predicting HGT insertion sites in different species as illustrated in Fig. 4. AUC values for species *Acidovorax sp. SD340*, *Mycolicibacterium monacense* and *Streptomyces griseofuscus* approach to 1.

### DeepHGT learns palindromic pattern at HGT insertion sites

The Repetitive Extragenic Palindromic (REP) sequence including an inverted repeat may have single or multiple adjacent copies [48]. A DNA sequence is considered as palindromic if it is equal to its reverse complement. For example, the DNA sequence CGTTGGCAACG is palindromic because its complement is GCAACCGTTGC, and reversing the complement gives CGTTGGCAACG, which is equal to the original sequence. The palindromic pattern makes REP sequences specific targets for some insertion sequence elements such as ISPsy8 in *Pseudomonas syringae* DC3000, ISPa11 in *P. aeruginosa* PA01, and so on [49]. This also demonstrates that REP elements are hot spots for specific transposition [50], which plays an important role in HGT, and contribute to genome instability, bacterial evolution, and speciation [51].

To test whether DeepHGT has learned sequence patterns such as palindromic subsequences as a significant local feature to recognize HGT insertion sites. First, we should measure the importance of palindromic patterns in the prediction process. We choose an optimal threshold to select correctly classified positive samples from the test dataset since the prediction value made by DeepHGT for each test sample is between 0 and 1. The optimal threshold in our experiment is set as 0.4935, which is calculated according to Youden’s J statistic [52]. From the correctly classified positive samples, each of which has true label as 1 and prediction value larger than the optimal threshold, we find 125 samples containing palindromic subsequence *S*_*palin*_={ *s*_*i*_, *i*=1,...,125}, such as the palindromic sequence TAAAAAGATAAGTTGAATATTCAACTTATCTTTTTA in the positive sample AACAAGGAATTGGATTATAAAATTGTAAAAAGATAAGTTGAATATTCAACTTATCTTTTTATTTCACGTCTATTTATCTTAAAACCTATTTTTTCTTCTATTTCTTTTAGCTGATTTTCA. Then, for each sample *s*_*i*_ in *S*_*palin*_, we measure the importance of its palindromic subsequences in affecting the prediction result. By comparing palindromic subsequences to randomly selected subsequences, we could test whether palindromic subsequences are significant important local features learned by DeepHGT.

In general, deep learning models transform and combine local features, which are extracted from input data through convolution layers, to make a prediction. As for one DNA sequence, any continuous subsequence can be treated as a local feature. However, not all local features are of equal importance in determining the prediction result. Therefore, we define the HGT-Index (HI) to measure the contribution of local features to the prediction value of the sequence. For sequence *S*, we record its prediction value made by DeepHGT as *l*. Then for any local feature or local subsequence *f*_*i*_, we set the output of this local subsequence in the first convolutional layer as 0 and record the corresponding prediction value of the sequence as *l*_*i*_. So the HGT-Index *HI*_*i*_ for *f*_*i*_ is defined as follows,

1 $$ {HI}_{i} = \left|l-l_{i}\right|  $$

By setting the output of the local subsequence in the first convolutional layer as 0, DeepHGT could not learn any feature information from this region. The local subsequence could not contribute to the final prediction. It also does not have any coincide with other important sequence features learned by DeepHGT. So |*l*_0_−*l*_*i*_| measures the contribution of the local subsequence *f*_*i*_ to the prediction.

From each sample *s*_*i*_ in *S*_*palin*_, we randomly select one palindromic subsequence *f*_*i*_ and compute its HGT-Index $HI^{palin}_{i}$, the length of *f*_*i*_ is at least 10 bp. As a comparison, we randomly select a subsequence $f^{0}_{i}$ with equal length no matter it is palindromic. The HGT-Index of $f^{0}_{i}$ is $HI^{0}_{i}$. This generates two sets of HGT-Index *HI*^*palin*^=$\left \{HI^{palin}_{1},...,HI^{palin}_{125}\right \}$ and *HI*^0^=$\left \{HI^{0}_{1},...,HI^{0}_{125}\right \}$. The null hypothesis is that *HI*^*palin*^ and *HI*^0^ have identical average values, which denotes that palindromic subsequences and random subsequences are consistent with each other. We calculate the T-test for the means of *HI*^*palin*^ and *HI*^0^. The T-test result is t-statistic=2.65864, *P*-value=0.00835, which rejects the null hypothesis. Therefore, palindromic subsequences are significantly important local features learned by DeepHGT to make the prediction.

We collect 6 palindromic sequences from REP sequences found in [49]. In order to test whether the 6 palindromic sequences contribute significantly to the prediction of DeepHGT, for a palindromic sequence *s*_*palin*_, we randomly select a sequence *S* from our test data set and record its prediction value *l*, then we randomly select a subsequence *s* of *S* and replace it with *s*_*palin*_. Now we have a modified sequence *S*_*palin*_ containing *s*_*palin*_. We feed *S*_*palin*_ into DeepHGT and record prediction value *l*_*palin*_. The HGT-Index of *s*_*palin*_ is |*l*−*l*_*palin*_|. As a comparison, we generate a randomized DNA sequence *s*_*null*_. *s*, *s*_*palin*_, and *s*_*null*_ have equal length. We replace *s* with *s*_*null*_ to get another modified sequence *S*_*null*_. The prediction value of *S*_*null*_ is *l*_*null*_. So the HGT-Index of *s*_*null*_ is |*l*−*l*_*null*_|. We repeat these operations 5000 times and get two sets of HGT-Index *HI*_*palin*_={$\left |l^{1}-l^{1}_{palin}\right |,...,\left |l^{5000}-l^{5000}_{palin}\right |$} and *HI*_*null*_={$\left |l^{1}-l^{1}_{null}\right |,...,\left |l^{5000}-l^{5000}_{null}\right |$}. The null hypothesis is that *HI*_*palin*_ and *HI*_*null*_ have identical average values, which means that there is no difference between the palindromic sequence and a random sequence in affecting the prediction of DeepHGT. Table 3 illustrates the statistical tests of 6 palindromic sequences found in Insertion Sequence elements. As we can see the three palindromic sequences found in ISPa11, ISRm22, ISPpu9, and ISRm19 significantly contribute to the prediction of DeepHGT.

**Table 3:** Statistical test of 6 palindromic sequences in Insertion Sequence elements

Insertion Sequence elements	palindromic sequence	*p*-value
ISPsy8	TGCCGACGCAGAGCGTCGCA	0.4304
ISPsy8	GGACGCGGAGCGTCC	0.3625
ISPa11	GGCGATCGCGCGATCGCC	1.0934*e*^−10^
ISPpu9	GCGGGCTAACCCGC	5.9209*e*^−14^
ISRm22	CCTTCCCCCGCGCGGGGGAAGG	8.0776*e*^−7^
ISRm19	ACCTTTCCCCGAGCGGGCGAAG	0.0068

### Evaluation of DeepHGT in an independent set of metagenomic samples

**Fig. 5:**
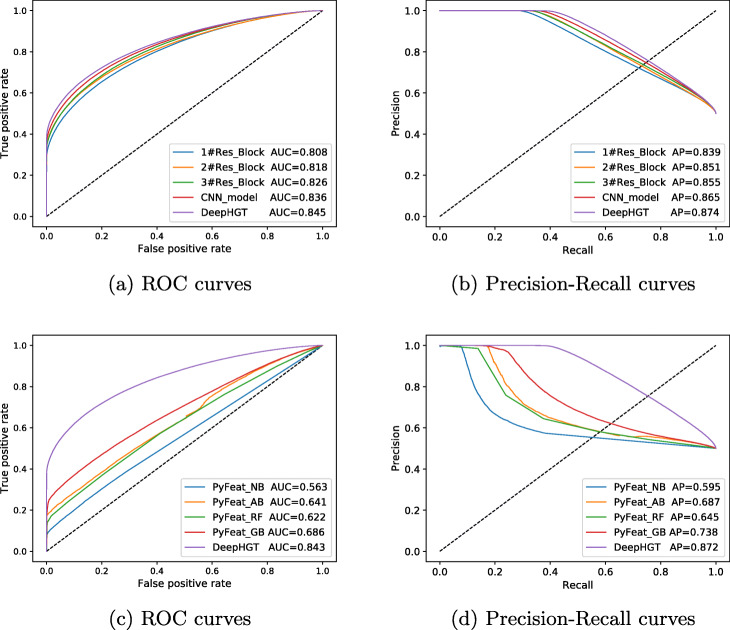
**a** and **b** compare ROC and Precision-Recall curves of DeepHGT and the other five deep learning models. DeepHGT has achieved the highest AUC value 0.8448 and AP value 0.8743. **c** and **d** compare the performance of DeepHGT and four machine learning models implemented in PyFeat

To further evaluate the generalization of DeepHGT, the set of 689,312 sequences obtained from 147 metagenomic samples [39] is used as an independent test. Figure 5a and b compare ROC and Precision-Recall curves of DeepHGT and the other five models. DeepHGT has achieved the highest AUC value 0.8448 and AP value 0.8743, which are a little lower than previous test results. Figure 5c and d compare the performance of DeepHGT with four machine learning models implemented in PyFeat. PyFeat_GB has achieved AUC value 0.686 and AP value 0.738 which are worse than DeepHGT. Table 4 compares the accuracy of DeepHGT and other methods. DeepHGT achieved the highest accuracy score of 0.762. As illustrated in Appendix Table 9, all pairwise Delong test results have a *p*-value of less than 0.05. So all models have significantly different performance. These experimental results demonstrate that DeepHGT has learned general sequence patterns that are shared by various HGT insertion sites on reference sequences. So DeepHGT could still achieve better performance than other models in this independent dataset. DeepHGT is a powerful model to accurately recognize HGT insertion sites.

**Table 4:** Comparison of accuracy of DeepHGT and other methods for an independent set of Metagenomic samples

1#Res_Block	2#Res_Block	3#Res_Block	CNN_model	DeepHGT
0.725	0.738	0.742	0.752	0.762
PyFeat_rf	PyFeat_ab	PyFeat_gb	PyFeat_nb	DeepHGT
0.580	0.588	0.604	0.500	0.762

### Some applications of DeepHGT

#### Likelihood of bacteria genomes harboring HGT insertions sites

Compared to LEMON, DeepHGT does not need next-generation sequenced (NGS) data as input. DeepHGT could recognize HGT insertion sites on raw DNA sequences according to sequence features. For reference bacteria genomes in NCBI, we could utilize DeepHGT to calculate their likelihood of harboring HGT insertions sites. For each reference, we use a 100 bp slide window to extract subsequences. The stride length is 50 bp. The subsequences are then feed into DeepHGT to get prediction values *P*={*P*_*i*_,*i*=1,...,*n*}. The likelihood of the reference is the mean value of *P*. Table 5 shows some references with high likelihood. These bacteria maybe more easily to receive adaptive advantages through HGT and are worthy of further research.

**Table 5:** Likelihood of harboring HGT insertions sites for reference bacteria genomes in NCBI

Genus	Accession	Likelihood	References
Streptomyces	NZ_JOJH01000630.1	0.535	[53, 54]
	NZ_LMFT01000033.1	0.997	
	NZ_LYOT01000881.1	0.999	
Mycobacterium	NZ_MVHE01000390.1	0.871	[55, 56]
	NZ_MVIC01000125.1	0.984	
	NZ_LZSE01000001.1	0.999	

#### Find bacterial genes enriched with potential HGT insertion sites

To find genes enriched with potential HGT insertion sites, we collect bacterial genes available from NCBI. For each bacterial gene, we use a 100 bp slide window on the gene region to extract subsequences. The stride length is 10 bp. The subsequences are then feed into DeepHGT to get prediction values *G*={*G*_*i*_,*i*=1,...,*n*}. The likelihood of the gene is $\bar {G}=\frac {1}{n}\sum _{i=1}^{n} G_{i}$. If $\bar {G}>0.5$, we regard the gene is enriched with potential HGT insertion sites. Finally, we collect 1,404 genes. We then perform Gene Ontology (GO) analysis for these genes. Table 6 shows some biologic processes associated with the most number of genes enriched with potential HGT insertion sites. As we can see 48 genes are associated with translation, whose efficiency is closely related to HGT [57]. Besides, 5 genes are associated with DNA integration, which is the integration mechanism of transferred genes.

**Table 6:** Biologic processes associated with the most number of genes enriched with HGT insertion sites

GO ID	Biologic Process	Gene count
GO:0006412	translation	48
GO:0005975	carbohydrate metabolic process	15
GO:0006355	regulation of transcription, DNA-templated	12
GO:0032259	methylation	10
GO:0009116	nucleoside metabolic process	7
GO:0045454	cell redox homeostasis	6
GO:0015074	DNA integration	5

#### Find potential hotspot of HGT insertion sites

By utilizing DeepHGT to scan reference bacteria genomes, we could find regions enriched with potential HGT insertion sites. For a reference sequence, we apply DeepHGT to calculate the distribution of potential HGT insertion sites over it. For each nucleotide of the reference, we extract a 100 bp subsequence, which has the nucleotide in the middle, as the input of DeepHGT. If the prediction probability is larger than 0.5, the nucleotide position is treated as one potential HGT insertion site. Then we slide a window over the reference sequence and utilize DeepHGT to get the number of potential HGT insertion sites in each window. The window size is *l*=100 and the number of potential HGT insertion sites in each window is *n*, then we use $r=\frac {n}{l}$ to measure the rate of HGT insertion sites in one sliding window. In our experiment, we set *r*>0.2 to filter out windows with low likelihood.

**Fig. 6:**
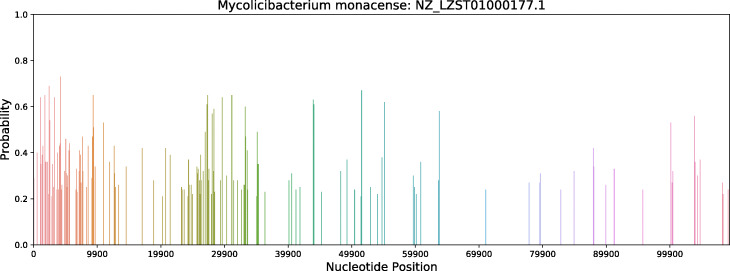
The distribution of potential HGT insertion sites predicted by DeepHGT over a reference sequence NZ_LZST01000177.1, which belongs to species *Mycolicibacterium monacense*

Figure 6 illustrates the distribution of potential HGT insertion sites predicted by DeepHGT over a reference sequence NZ_LZST01000177.1, which belongs to species *Mycolicibacterium monacense*. As we can see, regions with a high rate of potential HGT insertion sites are randomly distributed across the reference sequence. Some regions are close together. These regions are potential hot spots of HGT insertion sites and deserve more research to explore their relationship with HGT.

## Conclusion

In this paper, we propose a deep residual model named DeepHGT to predict HGT insertion sites on reference sequences. By utilizing LEMON, which is based on the traditional alignment technology to detect HGT breakpoints, we obtained two independent sets of sequence segments to train and test DeepHGT. On these two sets, DeepHGT outperforms PyFeat. Since DeepHGT recognizes HGT insertion sites on reference sequence according to sequence patterns, DeepHGT is not affected by sequencing coverage. So DeepHGT is a reliable model to recognize the HGT insertion site. It could help us detect potential HGT sites for further analysis. As we collect more HGT insertion sites and use them to train DeepHGT, it could learn more and accurate general sequence features about HGT insertion sites.

## Discussion

DeepHGT is the first deep learning model which could recognize HGT sites directly on bacterial genomes. DeepHGT is a very complicated model since it contains 2,119,297 trainable parameters. So, to make DeepHGT achieve powerful performance, we construct a very large data set to train DeepHGT. In our experiments, the main reason for DeepHGT achieving better AUC and AP values than other machine learning methods implemented in Pyfeat is that DeepHGT could learn more discriminant sequence features than the ones defined in Pyfeat. These features learned by DeepHGT should be treated as data-driven features. Furthermore, compared to LEMON, the main advantage of DeepHGT is that it need not paired-end DNA sequencing reads as input. So, by running DeepHGT on bacteria genomes and their coding genes available from NCBI, we could calculate the likelihood of bacteria genomes harboring HGT insertions sites and find bacterial genes enriched with potential HGT insertion sites. These preliminary results help us further research the mechanism, function, and benefit of HGT. This is also our future work.

## Methods

### Dataset

We collect bacterial reference sequences from the National Center for Biotechnology Information (NCBI) to construct a reference sequence set. It contains 109,419 bacterial reference sequences, which belong to 16,093 bacterial species. We index all references together to generate the Burrows-Wheeler Transform (BWT) indexes. LEMON is based on the traditional alignment method which takes shotgun metagenomic reads and the reference sequence set as inputs to detect and label HGT breakpoints. Based on the detected HGT breakpoints we collect 100 bp DNA sequences at HGT insertion sites on bacterial reference sequences. Each sequence has one HGT insertion site in the middle.

**Fig. 7:**
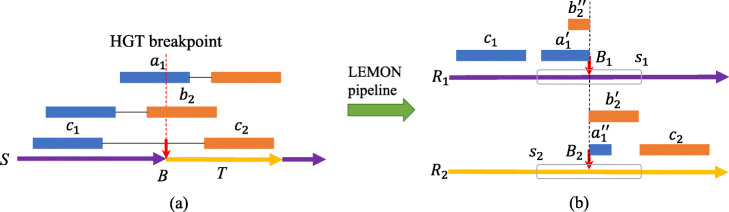
*S* is a sequenced strain, which contains one transferred segment *T* from reference sequence *R*_2_. *B* is a HGT breakpoint, which is supported by one paired-end read and two split reads *a*_1_ and *b*_2_. Through LEMON pipeline, we detect two HGT insertion sites *B*_1_ and *B*_2_ on references *R*_1_ and *R*_2_ respectively. For each split read, one portion of it is aligned to the left side of *B*_1_ and the other portion is aligned to the right side of *B*_2_. We then extract two 100 bp sequences *s*_1_ and *s*_2_ as input data of DeepHGT. *B*_1_ and *B*_2_ are in the middle of *s*_1_ and *s*_2_ respectively

As illustrated in Fig. 7a, *S* is a sequenced strain, which consists of a harbor sequence *R*_1_ and one horizontal transferred segment *T* from reference sequence *R*_2_. *B* is a HGT breakpoint on strain *S*, which is supported by one paired-end read and two split reads. Two split reads *a*_1_ and *b*_2_ are clipped on the *B*, which means that one portion $a^{\prime }_{1}$ of *a*_1_ is aligned to left side of *B*_1_, and the other portion $a^{\prime \prime }_{1}$ of *a*_1_ is aligned to right side of *B*_2_ as illustrated in Fig. 1b. Reads *c*_1_ and *c*_2_, which belong to one paired-end read, are on the two sides of *B*. Since HGT denotes the insertion of foreign gene, the sequences on two sides of *B* are belong to two different reference sequences, so *B* is corresponding to two breakpoint positions *B*_1_ and *B*_2_ on references *R*_1_ and *R*_2_ respectively as shown in Fig. 7b. We define *B*_1_ and *B*_2_ as HGT insertion sites. We then extract two 100 bp sequences *s*_1_ and *s*_2_, which have *B*_1_ and *B*_2_ in the middle respectively, as input data of DeepHGT. LEMON pipeline in Fig. 7 denotes the process of detecting HGT insertion sites from raw paired-end reads [34]. Firstly, we apply BWA software for mapping raw reads against the reference genomes. Then we utilize samtools to extract split reads and unique mapping reads. The mapping quality of unique reads is 20. The unique mapping reads and split reads are inputs of LEMON. LEMON utilizes paired-end reads to get candidate regions for HGT insertion sites and split reads to infer the precise insertion site positions on reference sequences. Each HGT insertion site is supported by at least one pair-end read and one split read. For example, the insertion sites *B*_1_ and *B*_2_ in Fig. 7b are supported by two split reads *a*_1_, *b*_2_ and one paired-end read *c*_1_−*c*_2_. Sequences *s*_1_ and *s*_2_ are treated as positive samples. To get negative samples, we randomly extract 100 bp DNA sequences from regions that are at least 10,000 bp away from the nearest HGT insertion sites on reference sequences. So there is no overlap between positive and negative samples.

### Data augmentation

**Fig. 8:**
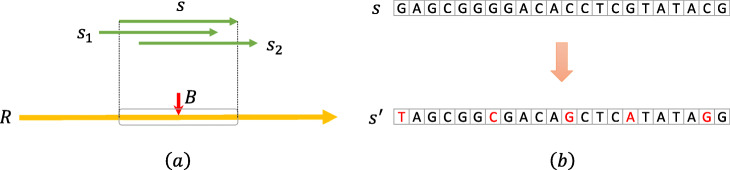
Two data augmentation methods. In (a), *R* is reference sequence. *B* is the HGT insertion site, which is in the middle of training sequence *s*. *s*_1_ and *s*_2_ are augmented training samples, which are sampled from near the HGT insertion site *B* within a small region. In (b), through randomly changing nucleotides in *s*, we get the augmented training sample *s*^′^, which retains most nucleotides of *s*

Data augmentation is an efficient technique to improve modern deep learning performance on image classification. Through a series of operations on images, the technique will expand the training set, which can aid deep learning models in learning robust features and achieve better performance. Therefore, to make DeepHGT fight overfitting and get better generalization in DNA sequence learning, we have tried two data augmentation methods as illustrated in Fig. 8. The first method in Fig. 8.a is shifting sampling positions near HGT insertion sites within a small region (±5*bp*) to get a vast amount of augmented training samples. Since the region is very small, the augmented samples contain most sequence information of HGT insertion sites. The second method in Fig. 8.b is to randomly change a small number of nucleotides for each training sample. The maximum number of nucleotides that are randomly changed is 10. Since most nucleotides of one sequence are retained, this method will not change the sequence pattern but increase the diversity of training samples, which helps DeepHGT to learn robust sequence features. The two augmentation methods are applied to all positive and negative training samples to generate augmented positive and negative training samples, which are used as the input of DeepHGT. In our experiments, these two techniques could improve around 0.01 ∼0.02 AUC value.

## Appendix

**Table 7:** The two percentage distributions of the top 10 most abundant species to which sequences in the positive training dataset and the independent positive test dataset belong

The positive training dataset	Percentage (%)	The independent positive test dataset	Percentage (%)
Microbacterium esteraromaticum	13.13	Faecalibacterium prausnitzii A2-165	7.69
Mycolicibacterium monacense	7.36	Microbacterium esteraromaticum	4.84
Mycobacterium sp. 852002-51961_SCH5331710	3.08	Prevotella copri DSM 18205	4.38
Faecalibacterium prausnitzii A2-165	2.39	Mycobacterium sp. 852002-51961_SCH5331710	3.5
Collinsella aerofaciens ATCC 25986	1.97	Mycolicibacterium monacense	3.04
Collinsella sp. 4_8_47FAA	1.94	Bacteroides stercoris ATCC 43183	2.53
Gemmiger formicilis	1.69	Roseburia faecis	2.33
Collinsella sp. TF06-26	1.64	Roseburia intestinalis L1-82	2.01
Bifidobacterium longum	1.55	Gemmiger formicilis	1.56
Bacteroides caccae	1.50	Acinetobacter sp. AR2-3	1.48

**Table 8:** Pairewise Delong test on AUCs of DeepHGT and other methods for test data set

	1#Res_Block	2#Res_Block	3#Res_Block	CNN_model	DeepHGT
1#Res_Block	–	<2.2*e*^−16^	<2.2*e*^−16^	<2.2*e*^−16^	<2.2*e*^−16^
2#Res_Block	–	–	0.000239	<2.2*e*^−16^	<2.2*e*^−16^
3#Res_Block	–	–	–	<2.2*e*^−16^	<2.2*e*^−16^
CNN_model	–	–	–	–	<2.2*e*^−16^
DeepHGT	–	–	–	–	–
	PyFeat_AB	PyFeat_GB	PyFeat_NB	PyFeat_RF	DeepHGT
PyFeat_AB	–	<2.2*e*^−16^	<2.2*e*^−16^	<2.2*e*^−16^	<2.2*e*^−16^
PyFeat_GB	–	–	<2.2*e*^−16^	<2.2*e*^−16^	<2.2*e*^−16^
PyFeat_NB	–	–	–	<2.2*e*^−16^	<2.2*e*^−16^
PyFeat_RF	–	–	–	–	<2.2*e*^−16^
DeepHGT	–	–	–	–	–

**Table 9:** Pairewise Delong test on AUCs of DeepHGT and other methods for an independent set of Metagenomic samples

	1#Res_Block	2#Res_Block	3#Res_Block	CNN_model	DeepHGT
1#Res_Block	–	<2.2*e*^−16^	<2.2*e*^−16^	<2.2*e*^−16^	<2.2*e*^−16^
2#Res_Block	–	–	<2.2*e*^−16^	<2.2*e*^−16^	<2.2*e*^−16^
3#Res_Block	–	–	–	<2.2*e*^−16^	<2.2*e*^−16^
CNN_model	–	–	–	–	<2.2*e*^−16^
DeepHGT	–	–	–	–	–
	PyFeat_AB	PyFeat_GB	PyFeat_NB	PyFeat_RF	DeepHGT
PyFeat_AB	–	<2.2*e*^−16^	<2.2*e*^−16^	<2.2*e*^−16^	<2.2*e*^−16^
PyFeat_GB	–	–	<2.2*e*^−16^	<2.2*e*^−16^	<2.2*e*^−16^
PyFeat_NB	–	–	–	<2.2*e*^−16^	<2.2*e*^−16^
PyFeat_RF	–	–	–	–	<2.2*e*^−16^
DeepHGT	–	–	–	–	–

## Data Availability

262 Metagenomic samples were deposited to Sequence Read Archive (BioProject: PRJNA475246). 147 Metagenomic samples were deposited to Sequence Read Archive (BioProject:PRJNA389280). Codes, training and test samples are freely available at https://github.com/lichen2018/DeepHGT.

## Abbreviations

AB: Adaboost Classifier
AUC: Area under the Curve of ROC
AP: Average-Precision
BWA: Burrows-Wheeler Alignment
BWT: Burrows-Wheeler Transform
BN: Batch Normalization
CNN: Convolutional Neural Network
DBSCAN: Density-Based Spatial Clustering of Applications with Noise
GB: Gradient Boosting
GO: Gene Ontology
HI: HGT-Index
HGT: Horizontal Gene Transfer
ICEs: integrative conjugative elements
MGE: Mobile genetic elements
NB: Naive Bayes
NCBI: National Center for Biotechnology Information
NGS: Next-Generation Sequencing
REP: Repetitive Extragenic Palindromic
RF: Random Forest
ROC: Receiver Operating Characteristic
SGD: Stochastic Gradient Descent
